# Shipwreck ecology: Understanding the function and processes from microbes to megafauna

**DOI:** 10.1093/biosci/biad084

**Published:** 2023-12-19

**Authors:** Avery B Paxton, Christopher McGonigle, Melanie Damour, Georgia Holly, Alicia Caporaso, Peter B Campbell, Kirstin S Meyer-Kaiser, Leila J Hamdan, Calvin H Mires, J Christopher Taylor

**Affiliations:** National Centers for Coastal Ocean Science, National Ocean Service, National Oceanic and Atmospheric Administration, Beaufort, North Carolina, United States; School of Geography and Environmental Science, Ulster University, Coleraine, Northern Ireland; Bureau of Ocean Energy Management, New Orleans, Louisiana, United States; Edinburgh Marine Archaeology, School of History, Classics, and Archaeology, University of Edinburgh, Edinburgh, Scotland, United Kingdom; Bureau of Ocean Energy Management, New Orleans, Louisiana, United States; Cranfield Forensic Institute, Cranfield University, Defence Academy of the United Kingdom, Shrivenham, England, United Kingdom; Woods Hole Oceanographic Institution, Woods Hole, Massachusetts, United States; School of Ocean Science and Engineering, University of Southern Mississippi, Ocean Springs, Mississippi, United States; Woods Hole Oceanographic Institution, Woods Hole, Massachusetts, United States; National Centers for Coastal Ocean Science, National Ocean Service, National Oceanic and Atmospheric Administration, Beaufort, North Carolina, United States

**Keywords:** artificial habitat, archaeology, experimental network, maritime cultural heritage, underwater cultural heritage

## Abstract

An estimated three million shipwrecks exist worldwide and are recognized as cultural resources and foci of archaeological investigations. Shipwrecks also support ecological resources by providing underwater habitats that can be colonized by diverse organisms ranging from microbes to megafauna. In the present article, we review the emerging ecological subdiscipline of shipwreck ecology, which aims to understand ecological functions and processes that occur on shipwrecks. We synthesize how shipwrecks create habitat for biota across multiple trophic levels and then describe how fundamental ecological functions and processes, including succession, zonation, connectivity, energy flow, disturbance, and habitat degradation, manifest on shipwrecks. We highlight future directions in shipwreck ecology that are ripe for exploration, placing a particular emphasis on how shipwrecks may serve as experimental networks to address long-standing ecological questions.

An estimated three million shipwrecks exist worldwide (UNESCO 2017). These shipwrecks are submerged in a variety of environments, including freshwater, estuarine, and saltwater. They extend from shallow rivers and bays to enclosed seas and the deepest basins of the oceans (figure 1). Wrecked vessels represent an array of forms, ranging from small, simply constructed watercraft, such as dugout canoes and rafts, to larger sailing ships and watercraft powered by steam or petroleum fuel. Each vessel served a purpose, often transporting people, supplies, and other cargo around the globe to explore unknown seascapes, colonize faraway lands, facilitate commerce and merchandise exchange, harvest marine resources, or engage in warfare.

**Figure 1.**
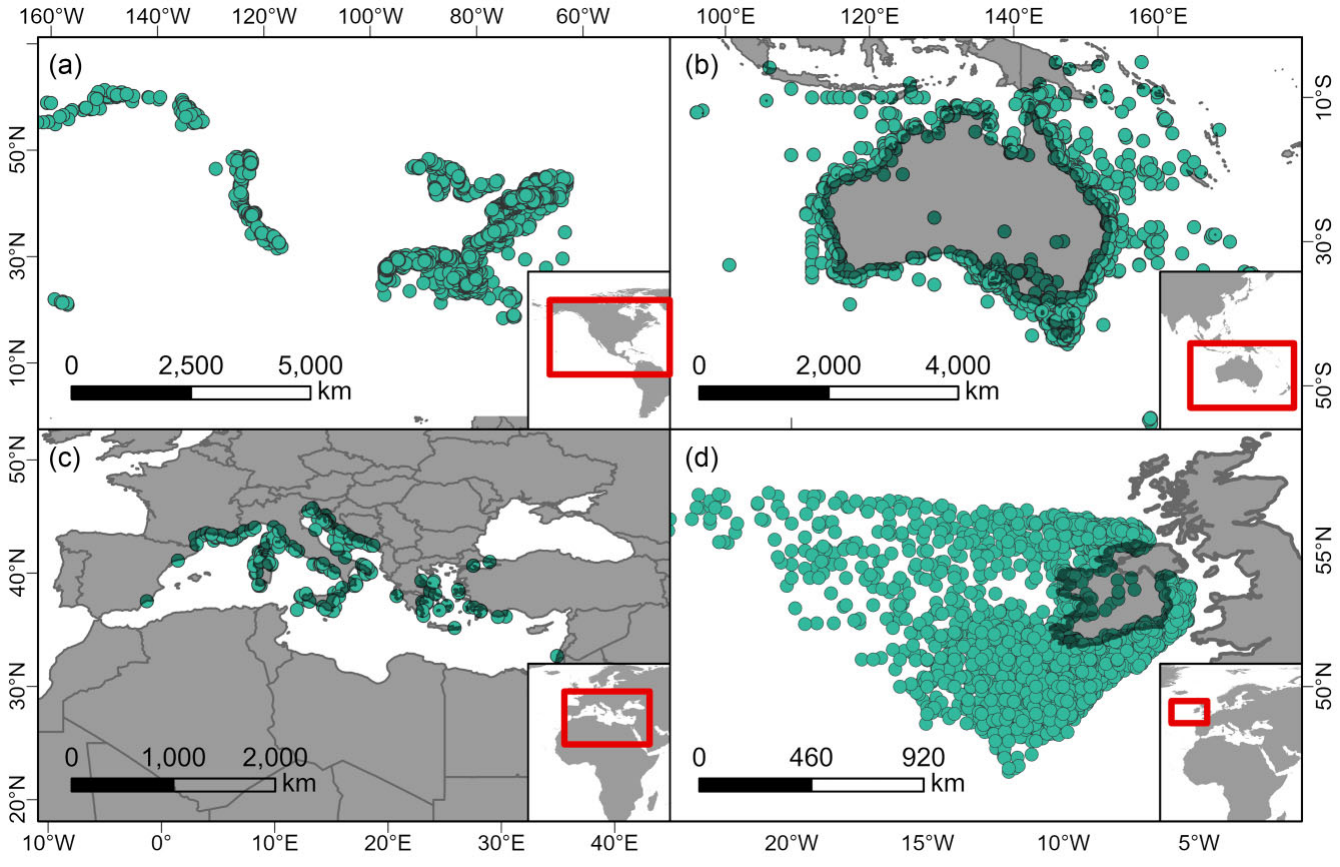
An estimated three million shipwrecks exist worldwide. Examples of potential shipwreck distributions by geographic region include (a) the United States (AWOIS 2016), (b) Oceania (Australian government 2021), (c) Mediterranean Sea up to CE 1500 (Strauss 2013), and (d) Ireland (Government of Ireland 2021). These regions are examples of shipwreck distributions from publicly available data sets in well-studied regions.

Vessels become shipwrecks when a catastrophic event occurs in a navigable water body. The catastrophe can be a natural event, such as a storm or hurricane, or can be human induced, such as running aground, capsizing, colliding (or alliding) with another vessel, scuttling, foundering, experiencing a mechanical or structural failure, or engaging in naval warfare, whereas derelict or intentionally sunk vessels are termed *abandonments*. Over time, wrecked vessels become submerged historical artifacts—representing tangible remains of past human behavior—and may also become foci of archaeological investigations that aim to illuminate the context and cultural legacy of an individual shipwreck within human history (Muckelroy 1978). Archaeologists seek to identify and describe the natural processes that break down shipwrecks over time, termed *site formation processes* (Muckelroy 1978). These physical, chemical, and biological processes interact with each other (Ward et al. 1999) and include effects from flora and fauna growing on shipwrecks and from moving water currents and underlying sediment.

Although shipwrecks are well known as cultural resources, they also form ecological resources because they may become habitat for a diversity of organisms. Lilian Lyle conducted the first published ecological study on shipwrecks when she surveyed macroalgal and invertebrate communities colonizing the German High Seas fleet sunk in Scapa Flow, Scotland (Lyle 1929). Among her pioneering discoveries, Lyle documented several new species colonizing the shipwrecks, pronounced vertical and horizontal ecological zonation patterns, and both similarities and differences between the ecological communities occupying shipwrecks and those on nearby rocky habitat (Lyle 1929). Since then, the number of ecological studies on shipwrecks has ballooned (supplemental figure S1) as ecologists harness opportunities for experimental and observational studies afforded by shipwreck habitats and archaeologists seek to better understand how shipwrecks are influenced by—and, themselves, influence—the aquatic environment.

In the present article, we build on this momentum and review shipwreck ecology as an emerging subdiscipline of ecology focused on understanding the ecological functions and processes that occur on and around shipwrecks. Because shipwrecks form both cultural and ecological resources, the subdiscipline is inherently interdisciplinary. The subdiscipline draws not only on long-standing links between archaeology and ecology but also on oceanography, limnology, chemistry, physics, geology, and engineering. We describe the burgeoning field of shipwreck ecology by synthesizing how shipwrecks create underwater habitats; how ecological functions and processes manifest, sometimes uniquely (and sometimes introducing ecological risks), on and around shipwrecks; and the relevance of shipwreck ecology to local communities and cultures. We conclude by outlining future directions in shipwreck ecology that are ripe for exploration and discovery and that hold promise for addressing long-standing, fundamental ecological questions.

## Shipwrecks provide underwater habitat for a diversity of life

When shipwrecks come to rest on the bottom, they encounter existing ecosystems that are structured (e.g., coral reefs, seamounts) or unconsolidated (e.g., gravel, sand, mud). Vessels have sunk on coral reefs (van der Schyff et al. 2020), seamounts (Ballard et al. 2018), rocky reefs (Bacci et al. 2016), lake beds (Caporaso 2017), river beds (Broadwater 1980), and—as in the case of ships including the infamous HMS *Titanic—*soft sediments in the middle of ocean basins. The introduction of a shipwreck to an existing ecosystem adds artificial structure and materials that can range from low relief (e.g., canoe, raft) to complex and vertically extensive (e.g., freighter, passenger liner). The added structure can be especially pronounced when the shipwreck lands on soft sediments that are otherwise devoid of hard structures, creating features that rise above the bottom and can be colonized by a host of organisms (Paxton et al. 2019a, Hamdan et al. 2021, Meyer-Kaiser et al. 2022b).

**Figure 2.**
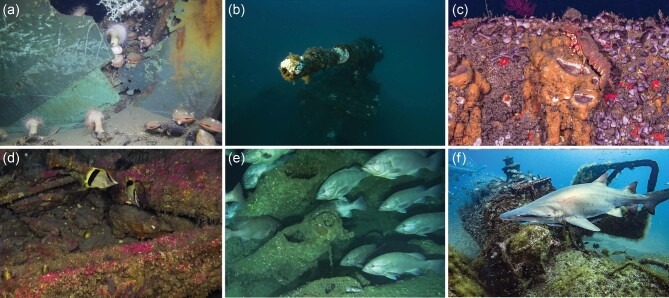
Shipwrecks form habitat for diverse marine life ranging from microbes to megafauna. Each panel depicts organisms found on shipwrecks throughout the world. (a) Microbes and sessile invertebrates colonize the nineteenth century wooden-hulled Ewing Bank shipwreck in 610 meters (m) in the Gulf of Mexico, in the United States. Photograph: Deep Sea Systems International/BOEM. (b) Sessile invertebrates including corals and hydroids colonize the deck gun of the World War II freighter *Hamlet* shipwreck in approximately 55 m in the Gulf of Mexico. Photograph: MITech/E. Kovacs. (c) Mobile invertebrates, including slipper lobster, are found on the ironclad shipwreck USS *Monitor* in 70 m off North Carolina, in the United States. Photograph: NOAA/Global Foundation for Ocean Exploration. (d) Cryptic fish species, including butterflyfish, inhabit the World War II tanker *E.* *M. Clark* in 80 m off North Carolina, in the United States. Photograph: NOAA/Global Foundation for Ocean Exploration. (e) Large demersal fish, including snowy grouper, hover above the conning tower of the German submarine U-*576* in 210 m off North Carolina, in the United States. Photograph: J. McCord/Coastal Studies Institute. (f) Sand tiger shark and reef fish glide above the ex-USS *Tarpon* shipwreck off North Carolina, in the United States. Photograph: T. Casserley, NOAA.

The artificial structure provided by shipwrecks creates underwater habitat for an array of flora and fauna ranging from microbes to megafauna (figure 2). The habitat provided by shipwrecks can be such a stark contrast to the surrounding sea-, river-, or lakebed that some shipwrecks have been termed *unique biodiversity hotspots* (Hamdan et al. 2021, Paxton et al. 2021). Shipwrecks provide substrate that microorganisms (Hamdan et al. 2021) and sessile benthic invertebrates (e.g., sponges, tunicates, cnidarians, bryozoans) grow on (Meyer et al. 2017). In euphotic zones, shipwrecks provide suitable substrate for micro- and macroalgae (Siciliano et al. 2016). Mobile benthic invertebrates, including crustaceans and echinoderms, occupy microhabitats afforded by shipwrecks (Meyer et al. 2017). Wrecked vessels also form havens for fish, including small cryptic species that reside within crevices, larger bottom-associated fishes that patrol the wreckage, and water-column associated baitfish and larger predators that school around and above shipwrecks (Ross et al. 2016, Paxton et al. 2021). Shipwrecks can support other macro- and megafauna, such as sea turtles (van der Schyff et al. 2020) and marine mammals (Arnould et al. 2015).

Shipwrecks can also negatively affect associated marine life. When shipwrecks sink, they land on natural habitats (e.g., sand, vegetation, reef) and can alter or destroy those habitats and their associated biota (Mancini et al. 2019, van der Schyff et al. 2020); such impacts have been well documented for ship grounding events on coral reefs (Schroeder et al. 2008, Raymundo et al. 2018). When shipwrecks reach the bottom, they provide substrate that may be suitable for and can facilitate the spread of invasive species that can alter reef communities, such as invasive coral (Soares et al. 2020) and corallimorphs (Work et al. 2008). Invasive species may also be transported by ships, such as in ballast water (Soares et al. 2022) and can potentially be released on wrecking. Shipwrecks potentially attract fauna (especially fish) from nearby natural habitats, reducing the number of fish on the natural habitats. There is an ongoing debate in the artificial reef literature on whether artificial structures attract (e.g., aggregate) fish from surrounding habitats or produce new fish biomass. Recent findings indicate that there is likely a spectrum of aggregation and production, where aggregation may occur initially followed by production over the long term (Layman et al. 2016, Layman et al. 2020). In addition, biota occupying shipwrecks may differ from those of natural habitats. For instance, if a shipwreck sinks in a sedimentary environment, organisms that are not often found in or on the sediment may colonize or associate with the wreck structure. This can lead to differences in ecological functions between shipwrecks and natural habitats (Simon et al. 2013, Ross et al. 2016, Paxton et al. 2020). Ultimately, this can change a natural habitat into a habitat composed of artificial structure. Such transformations can lead to shifts in ecological communities, such as when a shipwreck on a coral reef triggered a phase shift to an algae-dominated system (Kelly et al. 2012). Shipwrecks are distinct from artificial reefs because the latter are planned, prepared, and placed to achieve environmental management goals (Becker et al. 2018). Shipwrecks, on the other hand, may carry with them all cargo and materials on board, including oil, organic matter, and metal. This can lead to the release of environmental contaminants that alter the surrounding biological communities (Schiel et al. 2016).

Compared with other benthic habitats, shipwrecks have unique spatial distributions (figure 1). Examples from well-studied regions, including the United States (figure 1a), Oceania (figure 1b), the Mediterranean Sea (up to CE 1500; figure 1c), and Ireland (figure 1d), highlight the unique nature of shipwreck distributions. Unlike networks of rocky or coral reefs that can extend for multiple kilometers across the seafloor or oyster reefs that expand horizontally or vertically over time, shipwrecks are discrete features. With few exceptions, shipwrecks are small islands of structure in the seascape, and the amount of structure provided by the shipwreck can never increase but instead can either remain constant (e.g., preserved by anoxic conditions) or reduce over time through both biotic and abiotic degradation. Although some habitats such as coral reefs are restricted by latitudinal boundaries, shipwrecks span global latitudes and water depths. Shipwreck distributions can mirror patterns in human decisions and behaviors (e.g., port locations, trade routes, conflicts) and can reflect locations of extreme or variable physical (e.g., currents, winds) or geological (e.g., shoaled sand, narrow passes) conditions. There is still, however, a degree of randomness related to the location of individual wrecking events.

Temporally, shipwrecks exhibit some parallels to ephemeral ecosystems such as whale falls and hydrothermal vents. For instance, whale falls go through distinct successional phases, resulting in a prolonged reef phase (Smith et al. 2015), and hydrothermal vents can become inactive over time (Van Dover 2000). Similarly, shipwreck materials can degrade over time from physical, chemical, biological, and geological processes. Shipwrecks, like whale falls, also represent a pulse introduction of structure and organic material (Smith et al. 2015, Caporaso 2017). A key difference among these ephemeral ecosystems, however, is that shipwrecks are human-made structures, whereas whale falls are biological inputs, and hydrothermal vents are geological phenomena. The maximum time scales of these ephemeral features also differ, because whale falls generally persist on a scale of decades, shipwrecks on a scale of centuries, and hydrothermal vents on a scale of millennia (Hamdan et al. 2021). Furthermore, the time scales for shipwreck persistence can vary on the basis of factors that include depth, water temperature, and material.

## Fundamental ecological functions and processes manifest on shipwrecks

**Figure 3.**
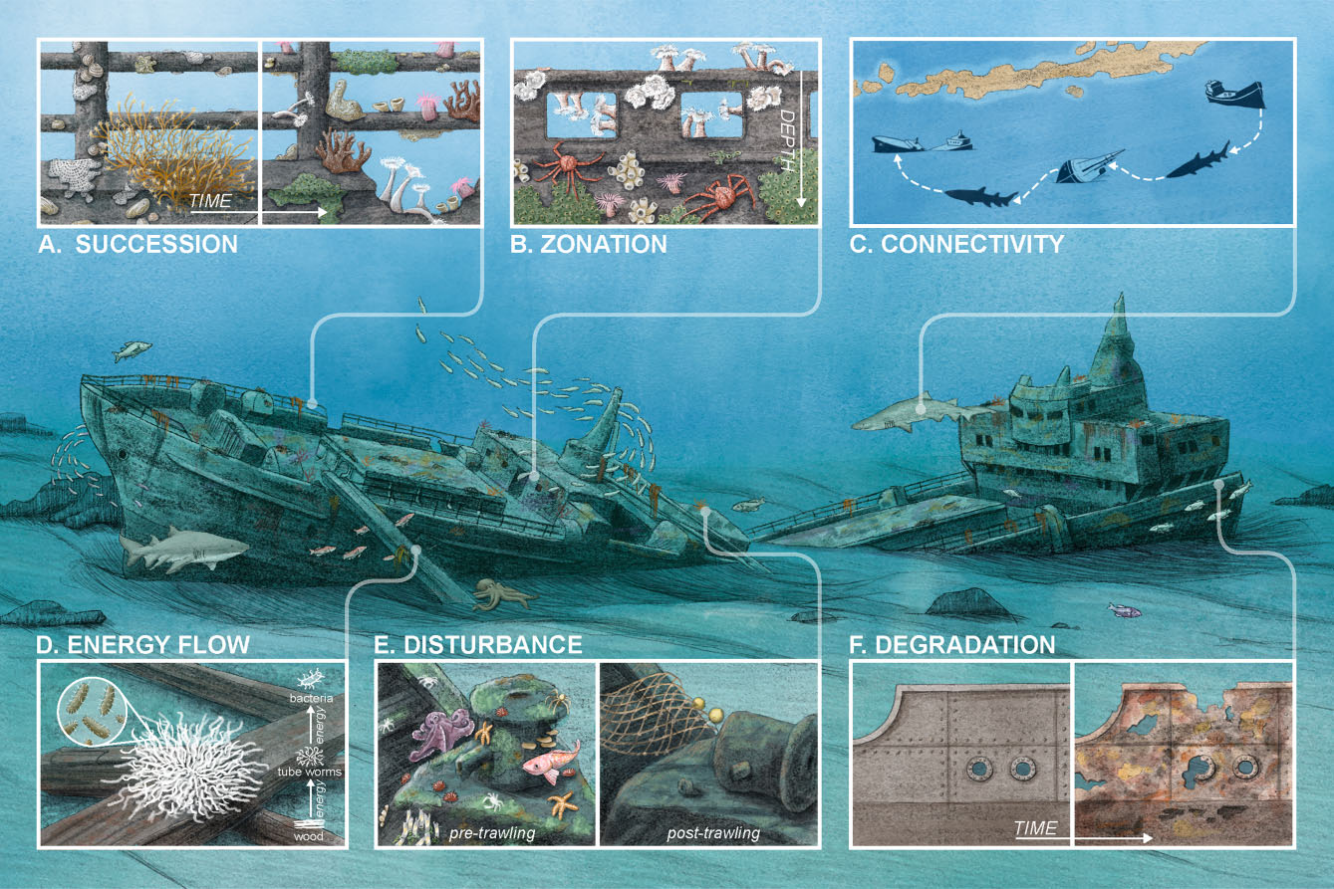
Fundamental ecological functions and processes occur on shipwrecks, including (A) succession, (B) zonation, (C) connectivity, (D) energy flow, (E) disturbance, and (F) habitat degradation. Each inset panel of the conceptual diagram depicts an example of one of the core ecological processes and functions that has been previously documented to occur on metal shipwrecks in particular ocean depths and geographic regions. (A) Succession where primary biofilm colonizers and other initial colonizers prepare shipwreck structure for secondary colonizers. (B) Zonation where large invertebrate suspension feeders are on upper (shallower) parts of shipwrecks with mobile and smaller encrusting invertebrates closer to the seabed. (C) Connectivity where shipwrecks act as stepping stones facilitating movement of fauna, such as sharks. (D) Energy flow where chemosynthetic bacteria support tubeworms growing on organic matter. (E) Disturbance where anthropogenic pressures, such as trawling, can alter shipwreck morphology and relief likely influencing habitat use. (F) Degradation of shipwreck structure over time via abiotic and microbially influenced corrosion. Illustration by Alex Boersma (www.alexboersma.com).

Foundational ecological functions and processes that have been documented in other natural ecosystems also occur in habitats created by shipwrecks (figure 3). In the present section, we review and describe how these processes manifest on and around shipwrecks.

### Succession

The succession of shipwreck ecological communities begins before the ships sink, because ships often carry biological materials or organisms, such as cargo provisions (e.g., food, drink), companion animals (e.g., livestock, pets), infestations or pests (e.g., rats, insects, mold, mildew), invasive species (in or on the vessel), marine growth, and biochemical products. For example, wooden sailing vessels often have teredo worm (*Teredo* spp.) infestation or barnacle growth on the parts of the vessel submerged below the water line. Antifouling measures, including the application of copper sheathing or specially formulated paints, help prevent the growth of those organisms and the subsequent impedance to sailing speed and maneuverability. Such antifouling measures may also continue to prevent organisms from growing on shipwrecks even following the sinking event, as in the case where the copper-sheathed wooden hull of a schooner was completely devoid of fauna more than a century after wrecking, likely because copper is toxic to most marine life and can deter invertebrate settlement (Hartland et al. 2019).

The process of wrecking can substantially and irreversibly alter the structure and structural integrity of a vessel (e.g., storm damage, artillery damage, collision damage, or an impact with the bottom). In most cases, the structural conditions and biological conditions of shipwrecks when they first come to rest on the lakebed, riverbed, or seafloor are unknown, with the exception that when a vessel sinks, any fouling fauna attached to the hull, as well as biological cargo, are introduced to the bottom. Depending on the sinking circumstances, these fouling organisms may be scraped off of the vessel during sinking, and some may be unable to survive at the depth where the shipwreck comes to rest. Other taxa may persist. On reaching the bottom, shipwrecks undergo a period of rapid change before reaching an environmental equilibrium, which is the state in which the rate of structural degradation by physical, chemical, and geological processes approaches zero (Muckelroy 1978). These archaeological site formation processes affect the state of the shipwreck over time and can be linked with ecological succession (Meyer-Kaiser and Mires 2022) or with the progressive shift in community composition over time from primary colonizers to an apex or climax community of semisteady state.

Ecological succession processes on shipwrecks are defined by the primary material the ship was made from and the environmental setting of the wrecking location (figure 3a). Wooden shipwrecks constitute large organic matter falls and can attract similar organisms to those observed at woodfalls, including opportunistic sessile or mobile invertebrates, such as burrowing mollusks (e.g., subfamily Xylophagainae; Turner 1973), whereas iron and steel shipwrecks are an ideal habitat for iron-dependent microorganisms (e.g., Zetaproteobacteria; McBeth and Emerson 2016, Price et al. 2020) and opportunistic invertebrates, such as bryozoans, tunicates, polychaetes, and crustaceans (figure 3a; González-Duarte et al. 2018). Environmental conditions also mediate succession on shipwrecks and can outweigh the differences in materials in dynamic environments (González-Duarte et al. 2018, Moseley et al. 2022).

Regardless of the shipwreck material or cargo, the primary colonizers of hard substrates introduced to the marine environment are microorganisms. These ubiquitous and opportunistic lifeforms play a keystone role in shipwreck succession because they rapidly respond to environmental disturbances and the appearance of new habitat features such as shipwrecks. Microorganisms initiate the biological steps of site formation when they attach to solid shipwreck components and form a community of cells and exopolysaccharides, called *biofilms* (Garrett et al. 2008). What microorganisms participate in the initial biofilm formation event is dictated by the physicochemical conditions of the site, including light, pH, nutrients, temperature, water depth, dissolved oxygen, and the properties of the hull and cargo materials (Grzegorczyk et al. 2018). As microbial populations accumulate within the biofilm, it reaches maturity (Lawes et al. 2016), at which point, cells detach and disperse through active, biologically mediated processes and passive physical disturbances (Toyofuku et al. 2016).

The exact sequence and time frame of succession on shipwrecks beyond biofilm formation depend on a range of factors and are largely unknown for many shipwrecks because they have often passed through early successional stages before they are discovered or studied. Future studies can aim to better document and understand the successional sequence and time frame on shipwrecks. Studies on intentionally deployed artificial reefs, ranging from ships to concrete modules, however, demonstrate that artificial habitats exhibit discrete successional stages, as is predicted by ecological theory, where the initial invertebrate settlers dominate and alter the substrate to make it suitable for tertiary colonizers (figure 3a; Perkol-Finkel and Benayahu 2005, Walker et al. 2007). Similarly, fish often colonize shipwrecks quickly, but because shipwrecks and other submerged watercraft are often discovered decades, centuries, or millennia after being lost, most information about colonization sequence on artificial substrates stems from intentionally deployed artificial reefs and observations that fish can appear within hours of deployment. The sequence of fish colonization varies, however, on the basis of geographic location and environmental conditions, such that either water-column-associated species, bottom-associated species, or a combination of both colonize initially (Dance et al. 2011, Paxton et al. 2018). Taken together, the findings on the fish colonization trajectory on artificial habitats suggests a mixture of attraction and production (Bohnsack 1989, Layman et al. 2020).

Although shipwreck communities undergo succession, communities on and around shipwrecks do not necessarily resemble established natural communities over time. This is largely driven by the shipwreck’s structure. Naturally occurring reefs are typically low lying, whereas shipwrecks, especially newly deposited vessels, are large, complex structures with high vertical relief and composite materials (Paxton et al. 2017). These differences in structure attract different communities of recruits, provide different microhabitats, and can lead to different community composition between shipwrecks and natural reefs even after a century (Perkol-Finkel et al. 2005, Perkol-Finkel and Benayahu 2007). However, although the community composition is different, species richness and diversity can be higher on structurally complex shipwrecks than on natural reefs (Meyer-Kaiser et al. 2022b). This has both positive and negative effects: Shipwrecks can lead to a net increase in regional biodiversity, but they can also harbor invasive species or facilitate range expansions (Paxton et al. 2019b, Soares et al. 2020). As a shipwreck degrades over time, the range of available microhabitats shifts, leading to changes in the community (Mires and Meyer-Kaiser 2023). Many shipwrecks are reduced to a pile of ballast stones or cargo, which represents the closest analogue to natural reefs. Much more research is needed to investigate the time scales for shipwreck communities to resemble natural reefs and especially the interactions between archaeological site formation and ecological succession over time (Meyer-Kaiser and Mires 2022). One study from the Mediterranean provides an endmember for understanding succession: Regional species composition was reflected in the community on a bronze battle ram from the First Punic War in 241 BCE (Gravina et al. 2021).

### Spatial zonation

Biota exhibit spatial zonation patterns on and around shipwrecks (figure 3b), as they do in other ecosystems, such as rocky shores. Microbial communities show predictable responses in biodiversity and composition in the sediments surrounding shipwrecks (Hamdan et al. 2021, Hampel et al. 2022a), largely in response to substrate availability (Hampel et al. 2022a, Moseley et al. 2022). In the Gulf of Mexico, for example, microbiomes (bacteria and archaea) in sediments near two deepwater wooden shipwrecks contained elevated taxa associated with organoheterotrophy and cellulolytic metabolism compared with the surrounding seafloor, signaling that shipwrecks subsidize carbon to seabed microbiomes long after their arrival (Hampel et al. 2022a). Predictable patterns in microbiome richness and diversity have been observed at a standoff distance from both wooden- and metal-hulled shipwrecks in marine settings (Hamdan et al. 2021, Hampel et al. 2022b), suggesting that shipwrecks shape the distributions of microorganisms across space and time and provide unique habitats for distinct taxa that are different from the surrounding environment. At finer scales, zonation can occur over a single shipwreck. For example, samples analyzed from the shallow estuarine Pappy Lane shipwreck revealed distinct microbial communities on portions of the wreck visibly corroded compared with areas without visible corrosion, likely reflecting selective pressure on microbiomes based on the vessel’s chemical composition or the surrounding environmental conditions (Price et al. 2020).

In addition to microorganisms, invertebrates also display vertical zonation, with high densities of large, sessile suspension feeders on the upper (e.g., shallower) areas of a shipwreck and mobile and smaller invertebrates and encrusting species nearer the seafloor (figure 3b; Meyer-Kaiser et al. 2022b). For example, dense clusters of filter-feeding anemones (*Metridium senile*) have been observed on upper portions of the shipwreck *Portland*, such as the walking beam (Meyer-Kaiser et al. 2022b). These patterns are largely driven by flow, because elevation in the benthic boundary layer facilitates suspension feeding (Vogel 1996). There are also horizontal patterns in invertebrates, with higher infauna abundance near shipwrecks than on the surrounding seafloor (Balazy et al. 2019).

Fish that occupy different depths zones of the water column associate with shipwrecks, which leads to additional zonation patterns. Typically, baitfish and large, pelagic predators (e.g., Carangidae, Scombridae, Sphyraenidae) occupy the water column above and around shipwrecks, whereas large demersal fishes (e.g., Serranidae, Lutjanidae) often reside near shipwreck locations closest to the bottom (Paxton et al. 2020). Smaller reef-associated fish occupy nooks and crannies within the shipwreck, also producing zonation patterns (Paxton et al. 2019b). In some instances, zonation can lead to differences in fish species richness and abundance in different areas of the water column. For example, on a shipwreck in the Mediterranean Sea, the majority of fish species (e.g., *Anthias anthias*, Clupeiformes) were located on the upper (shallower) zone of the shipwreck, and fewer were on the lower (deeper) zone (Sinopoli et al. 2015).

### Connectivity

Shipwrecks can facilitate ecological connectivity by forming stepping stones or connectivity corridors across the seascape (figure 3c), and this effect can be particularly pronounced in habitat-limited areas or in locations where shipwrecks are concentrated (e.g., Scapa Flow, Truk Lagoon). Microbial connectivity among shipwrecks is not fully understood, but evidence suggests that shipwrecks exert an island effect with elevated microbiome diversity closer to the wreck than the surrounding benthic and pelagic habitats. Islands or island-like systems reach biotic equilibria, where immigration rates of taxa equal extinction rates, with a resulting decline in alpha diversity with distance from the islands. Natural benthic habitat features exhibit this decline in diversity with distance, and it has also recently been documented with shipwreck-associated sediment and biofilms (Hamdan et al. 2021, Hampel et al. 2022b, Moseley et al. 2022). This island effect may also signal the role shipwrecks play in microbiome dispersal, especially where biofilms are concerned (Moseley et al. 2022). Some microbial taxa, such as those involved in biofilm formation on specific materials, may be more susceptible to shipwreck-assisted dispersal. Dispersal is a key stage in the lifecycle of a biofilm; it is therefore reasonable to expect that the selection of taxa would be based on material types, as well as the ability of taxa to reach new substrates (Moseley et al. 2022). Hydrodynamic modeling and particle tracking suggest that currents also mediate the dispersal of microorganisms across vast distances along the seabed (Hamdan et al. 2013).

Invertebrates exhibit connectivity among shipwrecks, because their larvae can use shipwrecks as stepping stones for dispersal (Meyer-Kaiser et al. 2022a). The larval dispersal of sessile invertebrates can be strongly influenced by oceanographic currents. Shipwrecks form substrates where larval invertebrates can settle, mirroring classic island biogeography patterns. For example, shipwreck size positively correlates with invertebrate species richness, and the distance among wrecks influences similarity in species composition (Meyer et al. 2017). Larval use of shipwrecks as stepping stones for dispersal and ultimately settlement can increase regional biodiversity (Meyer-Kaiser et al. 2022b), but it can also facilitate the dispersal of invasive species. World War II–related shipwrecks, for instance, facilitated the dispersal of the invasive cup coral (*Tubastraea tagusensis*) along Brazil's northern coast (Soares et al. 2020), and similar facilitation of invasions has occurred on intentionally deployed artificial reefs (Pinochet et al. 2020).

Artificial habitats, including shipwrecks, can facilitate the movement of reef-associated fish at the edges of climate ranges (Paxton et al. 2019b) and large predators (e.g., *Galeocerdo cuvier, Carcharias taurus*; figure 3c; Ajemian et al. 2020, Paxton et al. 2020) and can also promote occurrence of rare fish species (Medeiros et al. 2021). Shipwrecks can also facilitate the movement of or the spread of invasive fish species (*Pterois volitans*; Whitfield et al. 2006), potentially causing deleterious impacts. Some fish species move among habitats in the seascape but exhibit high site fidelity to shipwrecks. For example, Atlantic cod (*Gadus morhua*) return to the same shipwrecks following foraging excursions (Karlsen 2011), and sand tiger sharks (*Carcharias taurus*) return to the same or nearby shipwrecks (Paxton et al. 2019c). Marine mammals, such as fur seals (*Arctocephalus pusillus doriferus*), have also been documented to move among artificial habitats, including shipwrecks, while foraging (Arnould et al. 2015).

### Energy flow

The introduction of shipwrecks often creates opportunities for energy flow through pathways such as benthic–pelagic coupling and chemosynthesis (figure 3d). Shipwrecks may create halo effects for microfauna, with enhanced sediment microbial diversity and richness up to 200–300 meters (m) from the wreck itself (Hamdan et al. 2021, Hampel et al. 2022a) and for macrofauna, as is evidenced by elevated biodiversity of benthic invertebrates. These observations hint that shipwrecks are sources of organic matter, especially in oligotrophic conditions (Hampel et al. 2022b) and that the extent of their organic matter provisioning may relate to physical processes (Hamdan et al. 2013) and their time on the bottom (Stieglitz 2013). Infaunal enrichment provides available food for bottom-dwelling organisms, facilitating benthic–pelagic coupling (Balazy et al. 2019). It is also conceivable that dense populations of sessile invertebrates colonizing shipwrecks could increase benthic–pelagic coupling, with secondary production by the benthic species drawing carbon out of the water column, but this has not been tested.

Pelagic pathways based on phytoplankton production fuel sessile invertebrate filter feeders and their predators, whereas benthic primary production can support macroalgae, grazers, and higher levels in the food web on artificial structures (Cresson et al. 2014). Zooplanktivory is another key process on artificial habitats that can support energy transfer from zooplankton upward through the food web (Champion et al. 2015) that likely applies to shipwrecks as well, given that consistent spatial patterns occur in zooplankton, planktivorous baitfish, and piscivorous predatory fishes on and around shipwrecks (Paxton et al. 2019a). More broadly, it is also hypothesized that the energy flow on shipwrecks and other types of artificial habitats may relate to dynamic environmental conditions, such as those where currents can transport organic matter (Rouse et al. 2020).

Tubeworm communities are known to support chemosynthetic bacteria and the invertebrates with which they live symbiotically. In the Gulf of Mexico (Caporaso et al. 2018) and the Mediterranean Sea (Dando et al. 1992, Gambi et al. 2011), several observations have been made of tubeworms growing on organic matter carried as cargo within shipwrecks, such as paper, cotton, and produce. One example in the Gulf of Mexico, the steel-hulled former luxury yacht *Anona*, however, contains several small tubeworm colonies growing on the ship's wooden deck and a large colony of tubeworms located inside the aft cargo hold (Damour 2014). This site demonstrates that tubeworms may not exclusively colonize a shipwreck's decaying organic cargo but may also colonize a wreck's wooden structure (figure 3d).

### Disturbance

Shipwreck habitats are subject to environmental and anthropogenic disturbances that can alter their ecological communities and functions (figure 3e). Archaeologists have long investigated the disturbance of shipwrecks as it relates to site formation processes that can transition a shipwreck in and out of environmental equilibrium (Muckelroy 1978, Ward et al. 1999). The degree of disturbance relates to environmental conditions; shipwrecks resting in dynamic environmental conditions experience higher rates of disturbance. For example, Irish Sea shipwrecks in sand-dominated seascapes with high currents experience migrating waves of sediment across sites that trigger scour pits and alternating sediment deposition and erosion, whereas shipwrecks in mixed substrate or gravel-dominated settings experience less physical disturbance (Majcher et al. 2021). Climate-induced changes in environmental conditions, such as sea-level rise, warming water temperatures, and ocean acidification, are also hypothesized to disturb shipwrecks and potentially increase their degradation rate (Gregory et al. 2022). With the increasing frequency and intensity of storms and coastal hazards, shipwrecks face threats from pulse disturbances.

Human interactions with shipwrecks, although often integral for coastal community livelihoods, can also trigger disturbances. In shallow areas, shipwrecks form popular fishing and diving sites. Divers with poor buoyancy control or misplaced curiosity can physically disturb shipwreck structures and their associated organisms. In the Mediterranean, for example, divers disturbed macroalgal communities on the *Zenobia* shipwreck, altering its biotic cover (Siciliano et al. 2016). Fishing methods, such as trawling, over shipwrecks can physically change the wreck’s morphology and vertical relief, altering its habitat characteristics (Brennan et al. 2016). This can result in patterns where wrecks facing more fishing damage have decreased fish abundance and species richness compared with wrecks without fishing damage (figure 3e; Krumholz and Brennan 2015). The entanglement of ghost fishing gear is associated with lower abundance and different invertebrate community structure compared with unaffected portions of the same shipwreck (Meyer-Kaiser et al. 2022b, Mires and Meyer-Kaiser 2023), and ghost gear can continue to catch fish and other organisms (Laist 1995). Other types of disturbances can range from salvaging shipwrecks to reclaim portions of their cargo and obliterating shipwrecks that obstruct navigable waterways to removing dangerous materials (e.g., oil) from wrecks. Resource management plans that include an understanding of the network of ecological, sociocultural, and archaeological interactions that occur on shipwrecks can help maintain the sites and their value for the communities that rely on them.

Oil and other pollution spills can also disturb shipwrecks. The 2010 *Deepwater Horizon* oil spill in the Gulf of Mexico triggered new research on how biofilm and sediment microbiome exposure to external oil from the spill and associated chemical dispersants affected shipwreck preservation (Damour et al. 2016, Hamdan et al. 2018). Shipwrecks close to the spill origin were subjected to a newly observed phenomenon known as *marine oiled snow sedimentation and flocculent accumulation* (Passow et al. 2012, Hamdan et al. 2018), and the microbial communities surrounding the shipwrecks experienced sharp declines in microbial diversity compared with unaffected sites (Hamdan et al. 2018). The accompanying biofilm experiments revealed shifts in bacterial community structure and function, along with enhanced metal corrosion on steel samples (proxy for steel-hulled shipwrecks) exposed to oil and dispersant compared with control samples (Salerno et al. 2018, Mugge et al. 2019a, 2019b). Field observations confirmed oil-enhanced metal corrosion, demonstrating a rapid rate of hull deterioration at wrecks in benthic areas acutely affected by the spill in the 4 years following the oil spill compared with the 9 years prior (Damour et al. 2019).

Shipwrecks themselves can trigger disturbances to the natural habitats on which they sink. A large body of evidence on modern ship groundings details the impacts of grounding events on coral reefs. There is a key distinction between modern shipwrecks and historic shipwrecks. Historic shipwrecks, generally speaking, are those that sank more than 50 years ago (or more than 100 years in some nations), whereas modern shipwrecks sank more recently. In the Line Islands, for example, debris from modern shipwrecks composed of iron triggered rapid phase shifts within as little as 3 years of grounding events, resulting in black reefs (Kelly et al. 2012). These black reefs were dominated by algae and cyanobacterial mats; coral cover decreased from 40%–60% to less than 10%. The water surrounding the modern shipwrecks appeared cloudy and dark. Algal tissue samples revealed iron levels six times higher than at nearby unaffected sites, and the microbial community contained virulence genes and pathogens. A modern shipwreck on an isolated Palmyra atoll also triggered a phase shift on the reef as the coral cover decreased and the densities of invasive corallimorphs (*Rhodactis howesii*) increased (Work et al. 2008). In such cases, the removal of the modern shipwrecks (e.g., derelict vessels, which are not categorized as historical shipwrecks in archaeological contexts) may be considered to prevent further harm to natural ecosystems (Work et al. 2008), but even after removal, environmental impacts can persist (Work et al. 2018). Historic shipwrecks, however, should not be removed because they have archaeological importance, provide information about past human behavior, and may contain human remains. In cases in which historic shipwrecks are or may someday release pollutants into the environment, those wrecks can often be remediated to carefully remove the threat or pollutants without the need to destroy or remove the entire wreck.

### Habitat degradation

Although disturbance can degrade shipwreck structure, biogeochemical processes and destruction from organisms colonizing shipwrecks can also cause habitat degradation (figure 3f). Deterioration processes are linked to the type of material, commonly wood or metal, originally used to construct the vessel. The degradation of wooden vessels is caused by bacteria, fungi, and wood-boring or wood-consuming organisms (Blanchette 2000). Bacteria rapidly colonize but slowly attack the wood through erosion, tunneling, or cavitation, all mediated by extracellular enzymes (Kim and Singh 2000). Fungi also produce extracellular enzymes that break down the cell walls of the wood, causing more rapid degradation relative to bacteria acting without a host. Some fungi (white- or brown-rot fungi) can persist in aerobic environments when they are not fully submerged (Blanchette 2000), whereas others (soft-rot type fungi) can survive in marine environments with low dissolved oxygen (Unger et al. 2001). Wood-boring mollusks, called *shipworms* (teredinids), have worm-like bodies and use their anterior bivalve shell to burrow into the exterior wood surface during their larval stage (Distel et al. 2017). Shipworms remain within the wood for the duration of their life span, depositing a calcium carbonate substance that forms distinctive tubes throughout the wood's interior structure. But other wood-boring mollusks, called *piddocks*, also burrow into wood. Because they do not directly consume wood particles (although bacterial symbionts are hypothesized to digest wood; Distel et al. 2017), piddocks cause damage to the surface rather than the interior of the wood like shipworms do (Turner 1973). Wood-boring crustaceans such as isopods hatch broods within wood substrates and excavate wood burrows using their mandibles, whereas amphipods widen existing isopod burrows, and decapods, such as squat lobster (*Munidopsis andamanica*), can consume wood (Hoyoux et al. 2009, Shipway 2013).

The corrosion of metal shipwrecks in marine environments is a biogeochemical process where ferrous materials such as iron and steel exposed to oxygen in highly conductive seawater combined with microbial biofilms create an electrochemical reaction (figure 3f; Little and Lee 2007, Beech and Campbell 2008). The corrosion process consists of different phases, where corrosion is initially rapid but then slows as its products and microbial biofilms thicken and reduce the availability of the oxygen needed for corrosion (Beech and Campbell 2008). Microbially induced corrosion, often mediated by sulfate-reducing bacteria in marine environments, can catalyze biotic corrosion processes, often functioning in tandem with abiotic corrosion processes (Little and Lee 2007). Investigations into metal shipwreck corrosion indicate that these rates are neither linear nor constant and vary substantially even among nearby sites (Moore 2015), largely because of environmental conditions, such as salinity, dissolved oxygen concentration, temperature, and pH, as well as the extent of hull damage (Moore 2015).

As shipwrecks degrade, some have the potential to release pollutants carried as cargo or used as fuel, particularly those constructed and used in the twentieth century. Their pollution potential varies on the basis of the vessel’s construction material, the cargo that the vessel carried when it sank, and the local environmental and human-induced factors that influence the wreck's structural integrity over time (e.g., storms, salvage; Carter et al. 2021). Some metal vessels leach heavy metals such as arsenic, copper, and lead as they corrode, which can influence nearby biota (Van Landuyt et al. 2022). Iron enrichment from shipwrecks, for example, induced phase shifts on coral reefs from coral to algal and cyanobacterial dominance (Kelly et al. 2012). In freshwater lakes, elevated toxic metal concentrations have been observed close to shipwrecks, and it has been hypothesized that zebra mussels (*Dreissena polymorpha*) attached to metal shipwrecks might contribute to the filtration and subsequent deposition of these toxins (LaValle et al. 1999). Ships carrying oil can potentially continue to release it even after sinking events, but this risk is based on factors such as the state of wreck degradation, the amount of oil the vessel was carrying, and the sinking circumstances (e.g., whether oil was released during sinking or remains inside the hull; Carter et al. 2021). The effects of pollutants from wrecks can be long-lasting; evidence of contamination can be detected in the microbial community even as much as 80 years following the wrecking event (Van Landuyt et al. 2022). Environmental risk assessment frameworks (Carter et al. 2021) help categorize the level of pollution risk that shipwrecks pose and offer the capacity for targeted study to understand more about toxin bioaccumulation.

## Future directions in shipwreck ecology

Shipwrecks provide global networks of artificial habitats that can be used to address long-standing ecological questions, especially when coupled with advanced technologies.

### Harnessing opportunistic, experimental networks

Wrecks serve as millions of long-term experiments (e.g., settlement tiles, novel structure) spanning annual to millennial time scales and replicated across a range of physical, chemical, and biological conditions. This experiment is being added to continually through the accidental introduction of new shipwrecks with a high degree of spatially independent replication. These experimental characteristics of shipwrecks are ideal for conducting ecological studies in aquatic environments. Specifically, shipwrecks have the capacity to form monitoring networks similar to established national and international monitoring efforts that have assisted in understanding ecological patterns in natural ecosystems. For example, the global seagrass monitoring network Zostera Experimental Network (http://zenscience.org) coordinates eelgrass research used in global comparative experimental analyses, such as those revealing mechanisms behind top-down control in seagrass systems (Duffy et al. 2015) and the capacity for eelgrass to store blue carbon (Röhr et al. 2018). In the United States, the National Ecological Observatory Network (NEON) established across terrestrial and aquatic sites has led to novel ecological insights (Balch et al. 2020) and has been resilient in the face of the COVID-19 pandemic (Robinson et al. 2022). Establishing regional, national, or global monitoring networks of shipwrecks similar to existing monitoring programs for natural habitats offers the capability to better understand how these islands of artificial habitat function. A monitoring network of shipwrecks would afford opportunities to further test the principles of island biogeography, succession, disturbance, and additional ecological functions and processes over time and space. For example, the sequence and timeframe of succession on shipwrecks is largely unknown and could be better understood through intensified monitoring efforts. Environmental impacts from shipwrecks, related to pollution and phase shifts can also be further evaluated through a network of experimental sites. In addition, given the increasing extent of built structures in marine environments (Bugnot et al. 2021), establishing a long-term monitoring program on shipwrecks would provide lessons learned that could be applied to other types of built structures.

With changing climate and ocean conditions, shipwrecks can also serve as sentinel sites (Peirano 2013) for detecting changes in biological communities relative to baseline conditions. Although sentinel sites have been designated in ecosystems such as salt marshes as locations for long-term monitoring capable of quantifying and understanding changes in ecosystem functions and processes over time (Kennish 2019), the idea of sentinel sites has not been broadly applied within the context of shipwrecks (but see Peirano 2013 and Hamdan et al. 2018). Because shipwrecks may form connectivity corridors, facilitating species at their range edges (Paxton et al. 2019b), as well as invasive species (Soares et al. 2020), it is likely wrecks could continue to provide a barometer for studying changes in species distributions and community composition. In particular, shipwrecks could offer insight into range expansions of fish and other species moving poleward as they track suitable environmental conditions. Such a network of sentinel sites could also contribute to synergies with archaeologists documenting impacts of climate change on underwater cultural heritage sites more broadly (Gregory et al. 2022).

Ecological processes and functions that occur on shipwrecks provide lessons learned for how intentionally installed marine built structures, such as oil and gas infrastructure, marine renewable energy infrastructure, artificial reefs, and hardened shorelines may function ecologically under projected increases in the footprint of hard marine structures (Bugnot et al. 2021, Hampel et al. 2022a). For instance, studies on invertebrate and fish community development on shipwrecks may be a useful analog for expected patterns on offshore renewable energy infrastructure, such as wind turbine foundations and their associated scour aprons. In addition, insights on how shipwreck communities respond to disturbance may be useful for guiding artificial reef design and siting decisions in areas prone to human-induced or environmental disturbances. By extension, principles learned from shipwrecks on how artificial structures with different design features (complexity, material, relief) relate to ecological communities may be used to guide nature-based and nature-inspired designs intended to green existing infrastructure or foster coastal resilience because of increasing frequency of extreme climatic events (Feagin et al. 2021). For example, understanding how shipwrecks in shallow coastal waters change over time with climatic stressors can guide decisions on how best to design hybrid infrastructure to help overcome these stressors.

The ecological functions of shipwrecks, like other types of maritime heritage and natural ecosystems, are intimately tied to people, especially those in coastal communities who depend on and derive natural and social benefits from shipwrecks (Holly et al. 2022) and should be recognized when studying the ecology of shipwrecks. For example, shipwreck sites can provide fishing areas that support small-scale and subsistence fisheries and that can act as targets for recreational and commercial fisheries (Firth 2018). Wrecks in shallow waters can boost coastal resilience by forming breakwaters, as in the case of concrete vessels sunk just offshore of a beach shoreline in Virginia, in the United States, that act as buffers to erosion (Kiptopeke State Park). In the same vein as forming sentinel sites, shipwrecks and other cultural heritage can be monitored by local communities as indicators of ecological change, which can be particularly prescient when combined with traditional and local knowledge (Holly et al. 2022). The local cultural significance of shipwrecks and the cultural knowledge gained from them can enhance social cohesion and stability (Henderson et al. 2021), support local economies through heritage tourism (Grussing 2009), and provide a sense of identity and place (Mires 2014), and they may also represent the final resting place of the crew. Collectively, the benefits that shipwrecks can provide to coastal communities play integral roles in the blue economy and sustainable development on local, as well as international scales (Trakadas et al. 2019, Holly et al. 2022), and require thoughtful consideration on how to best balance benefits derived from shipwrecks with resource conservation, management, and preservation goals (Papageorgiou 2018, Holly 2022). Each shipwreck site is unique, performed its own role in human history, and is a nonrenewable resource; therefore, the collection of scientific information from shipwreck sites must not damage them. Lessons learned from studying shipwrecks and their benefits to coastal communities can help society to make pragmatic, balanced decisions about active management, *in situ* preservation, and managed retreat, for example, within the context of future ocean governance and marine spatial planning.

### Catalyzing ecological discovery with remote sensing

Advanced technologies can be harnessed to collect scientific information from shipwrecks and move the subfield of shipwreck ecology forward by addressing long-standing ecological questions, detecting changes in ecological communities over time, and better understanding links between shipwrecks and local communities. An unknown number of lost shipwreck sites remain undiscovered in waterways across the globe, and geophysical mapping and remote sensing tools can help locate these sites so that they may be documented, characterized, and better understood. Targeted shipwreck mapping (multibeam echosounder, side-scan sonar, airborne lidar) can not only identify shipwrecks within the broader landscape but generate three-dimensional (3D) habitat models with centimeter-scale (photogrammetry) to millimeter-scale resolution (laser-line scanning, synthetic aperture sonar) and determine the extent of shipwreck structure concealed beneath the seafloor (subbottom profiler, magnetometer). The resulting habitat maps can be used to study ecological questions on shipwrecks. For example, multibeam bathymetry and multifrequency backscatter acquired from multibeam echosounders have been used in concert with current and wave sensors to determine fluid and geological dynamics around shipwrecks (Majcher et al. 2021), and split beam echosounders and laser-line scanners have been used to determine patterns in fishes’ use of shipwrecks (Paxton et al. 2019a, Johnson et al. 2020). Photogrammetry has been used not only to develop 3D models of shipwrecks but to measure cover and growth of benthic invertebrates (Olinger et al. 2019) and to assess risk for oil pollution stemming from shipwrecks (Carter et al. 2021).

Data sets from different sensors can be merged to yield maximum information on shipwrecks. For instance, Damour and colleagues (2019) combined 3D optical and acoustic scanning systems (3D laser-line scanning, 3D sonar scanning, multibeam echosounder) and compared with still camera images to quantify the extent of enhanced metal corrosion occurring on historic shipwrecks after a major oil spill. Biological observations can be made with a variety of optical instruments (e.g., drop cameras, stereo cameras, high-resolution video, low-light cameras, hyperspectral imaging) and acoustic instruments (e.g., passive acoustics, active acoustics), and with physical sampling instruments (e.g., environmental DNA, benthic samplers, nets). Shipwreck mapping and biological observations can be made using the aforementioned instruments operated off an array of platforms, ranging from vessels, remotely operated vehicles, autonomous underwater vehicles, human-occupied vehicles, autonomous surface vehicles, unoccupied aerial vehicles, and divers. The choice of which instruments and platforms to employ are often determined by site-specific factors, including water depth and physical environment (current, waves, temperature, etc.). Interdisciplinary collaborations with engineers, geologists, and archaeologists are necessary to continue to develop methods most appropriate for assessing shipwrecks in extreme environments and to ensure that surveys are designed to extract the maximum amount of data possible in support of ecological and archaeological inquiries.

## Conclusions

Shipwreck ecology recognizes the foundational research by and collaborations between archaeologists and ecologists. The subfield does not exist within a vacuum of either ecology or archaeology, but instead fuses these two fields to pursue a mechanistic ecological understanding of processes and functions that manifest on and around shipwrecks. The findings from shipwreck ecology therefore have implications not only for ecology but also for archaeology, similarly to how maritime or nautical archaeology, although they are rooted in archaeology, have biological and ecological implications. The subdiscipline thrives because of collaborations not only with archaeologists but also with geologists, physicists, chemists, and engineers, and there is ample room for growth and continued investigations drawing on interdisciplinary insights and hypotheses that can be used to further understand how living resources created by shipwrecks function. Although we focus in the present article on the ecology of shipwrecks, we recognize other types of underwater cultural heritage exist, including submerged settlements, aircraft, middens, and landscapes, which likewise support ecological resources (Meyer-Kaiser and Mires 2022). Collectively, cultural heritage offers a vast network of ecological experimental and monitoring sites that can help expand on our collective understanding of ecological functions of submerged human-built structures.

## Supplementary Material

biad084_Supplemental_File

## Author Biography

Avery B. Paxton (avery.paxton@noaa.gov) and J. Christopher Taylor are affiliated with the National Centers for Coastal Ocean Science, National Ocean Service, National Oceanic and Atmospheric Administration, in Beaufort, North Carolina, in the United States. Christopher McGonigle is affiliated with the School of Geography and Environmental Science at Ulster University, in Coleraine, Northern Ireland. Melanie Damour and Alicia Caporaso are affiliated with the Bureau of Ocean Energy Management, in New Orleans, Louisiana, in the United States. Georgia Holly is affiliated with Edinburgh Marine Archaeology, in the School of History, Classics, and Archaeology at the University of Edinburgh, in Edinburgh, Scotland, in the United Kingdom. Peter B. Campbell is affiliated with the Cranfield Forensic Institute, at Cranfield University, Defence Academy of the United Kingdom, in Shrivenham, England, in the United Kingdom. Kirstin S. Meyer-Kaiser and Calvin H. Mires are affiliated with the Woods Hole Oceanographic Institution, in Woods Hole, Massachusetts, in the United States. Leila J. Hamdan is affiliated with the School of Ocean Science and Engineering at the University of Southern Mississippi, in Ocean Springs, Mississippi, in the United States.
