# Supplementary material for: Shipwreck ecology: Understanding the function and processes from microbes to megafauna
Source: Bioscience. 2023 Dec 19;74(1):12–24. doi: 10.1093/biosci/biad084 (PMC10831220; doi:10.1093/biosci/biad084)
Supplement: biad084_Supplemental_File [file biad084_supplemental_file.docx]

**Supplemental Information**

**Title:** Shipwreck ecology: understanding the function and processes from microbes to megafauna

**Authors:** Avery B. Paxton^1*^, Christopher McGonigle^2^, Melanie Damour^3^, Georgia Holly^4^, Alicia Caporaso^3^, Peter B. Campbell^5^, Kirstin S. Meyer-Kaiser^6^, Leila J. Hamdan^7^, Calvin H. Mires^6^, J. Christopher Taylor^1^

**
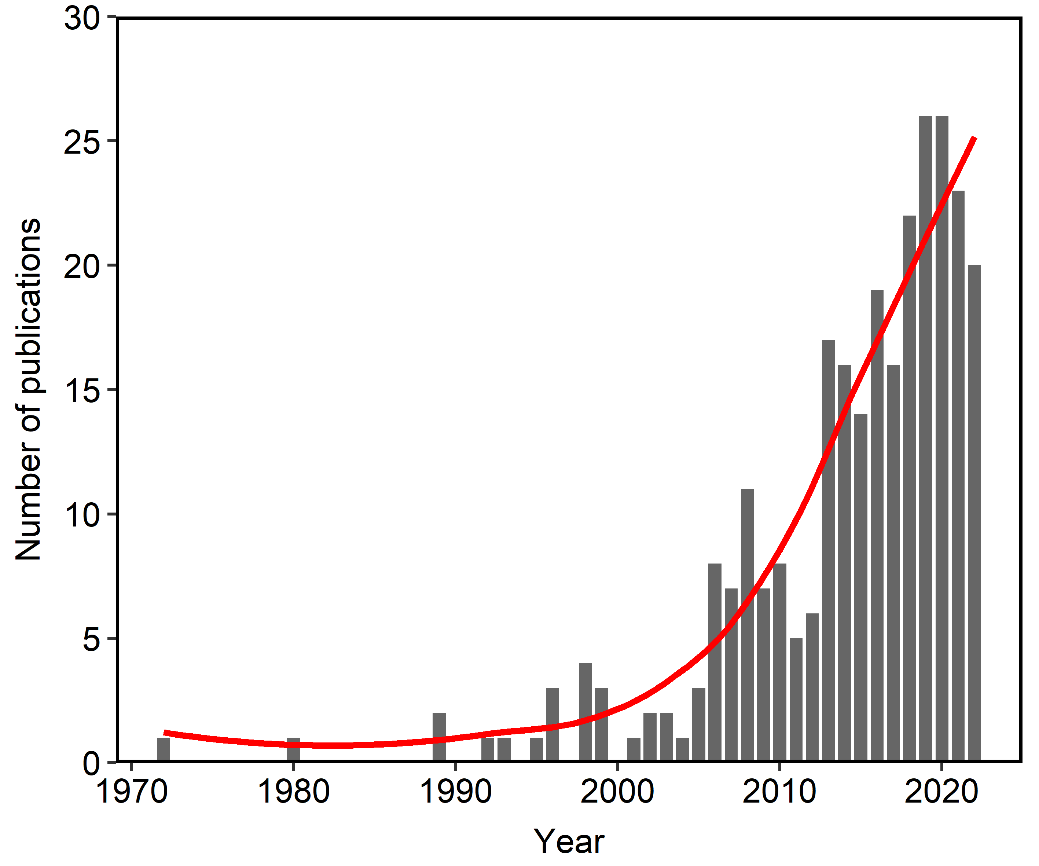
**

**Figure S1:** Number of peer-reviewed publications on shipwreck ecology or biology per year from 1972 through present. The 277 total publications were returned during a Web of Science search on 26 July 2022 using the search string ALL = (shipwreck*) AND ALL = (ecol* OR biol*). Red line is smoothed trend in publications by year (l).
